# The Influence of Different Hydrothermal Processes Used in the Preparation of Brussels Sprouts on the Availability of Glucosinolates to Humans

**DOI:** 10.3390/foods13182988

**Published:** 2024-09-20

**Authors:** Anna Sadowska-Rociek, Joanna Doniec, Barbara Kusznierewicz, Tomasz Dera, Agnieszka Filipiak-Florkiewicz, Adam Florkiewicz

**Affiliations:** 1 Department of Plant Products Technology and Nutrition Hygiene, Faculty of Food Technology, University of Agriculture in Krakow, 31-120 Krakow, Poland; joannadoniecur@gmail.com (J.D.); tomasz.dera@urk.edu.pl (T.D.); agnieszka.filipiak-florkiewicz@urk.edu.pl (A.F.-F.); 2 Department of Food Chemistry, Technology and Biotechnology, Chemical Faculty, Gdansk University of Technology, 80-233 Gdansk, Poland; barbara.kusznierewicz@pg.edu.pl; 3 Department of Food Analysis and Quality Assessment, Faculty of Food Technology, University of Agriculture in Krakow, 31-120 Krakow, Poland; adam.florkiewicz@urk.edu.pl

**Keywords:** brussels sprouts, glucosinolates, isothiocyanates, indoles, in vitro digestion

## Abstract

Cruciferous vegetables represent a valuable source of bioactive compounds. However, there is currently a deficiency of information regarding the extent to which these compounds remain bioaccessible to the body following thermal treatment and digestion processes within the digestive tract. Accordingly, the aim of this study was to evaluate the impact of heat treatment and in vitro digestion on the level of selected bioactive compounds in Brussels sprouts. The Brussels sprouts samples were subjected to a range of thermal processing techniques, which were then followed by a simulated in vitro digestion. The investigated compounds were analyzed using UV-Vis spectrometry and liquid chromatography coupled with mass spectrometry (LC-MS). The findings revealed that the sous-vide method of cooking Brussels sprouts resulted in significantly higher losses of glucosinolates (GLS) in comparison to conventional cooking methods. No significant differences were observed with regard to isothiocyanates and indoles. The analysis of GLS following digestion revealed that the process was more effective after sous vide and traditional cooking, and slightly less effective after steam cooking. With regard to individual compounds, glucoraphanin (GRA), glucoraphenin (GIV), and gluconasturtiin (GNS) were found to be completely degraded, whereas methoxyglucobrassicin (metGBS) was the most resistant to digestion in both the sous vide and steamed Brussels sprouts. The results indicated that the process of simulating digestion had no significant impact on isothiocyanates and indoles. This suggests that, if present in the heat-treated samples, these compounds remained stable during the in vitro digestion procedure.

## 1. Introduction

The *Brassica* vegetables are a globally consumed food crop, comprising numerous species and varieties, including kale, broccoli, cabbage, cauliflower, and Brussels sprouts [1]. The consumption of *Brassica* vegetables has been associated with a reduced risk of developing chronic diseases, including cardiovascular disease, type 2 diabetes, obesity, and possibly certain types of cancer [2]. Among plant foods, cruciferous vegetables have been increasingly included in dietary recommendations due to their capacity to deliver health-promoting nutrients and compounds, with glucosinolates (GLS) representing a particularly notable example. 

Although glucosinolates themselves lack direct health-beneficial properties, they can be converted by the enzyme myrosinase, which is naturally present in plants, into other compounds that may have beneficial effects on human health. This conversion occurs when the glucosinolates are broken down by chewing or cutting raw vegetables [1,3]. The hydrolysis products include, among others, isothiocyanates, thiocyanates, nitriles, and indoles, which are biologically active compounds with anticancer properties [2]. Among these compounds, isothiocyanates and indoles are of particular importance due to their health benefits [4,5]. 

The majority of cruciferous vegetables, particularly Brussels sprouts (*B. oleracea* (L.) var. *gemmifera*), are rarely consumed raw and require further heat treatment prior to ingestion. Conventional cooking methods, including steaming and the utilization of novel processing techniques such as hydrothermal sous-vide treatment, have been demonstrated to significantly influence the biochemical composition of cruciferous vegetables [6,7]. Nevertheless, research based on food composition alone is insufficient to determine the concentrations of individual components that are ultimately accessible to the human body following digestion. Following digestion in the human digestive tract, vegetable products are subjected to a range of conditions (e.g., pH changes, enzymes, electrolytes, and mechanical disintegration), which can impact the stability and bioaccessibility of their compounds. Consequently, the bioaccessibility of *Brassica* vegetables must be taken into consideration when evaluating their nutritional value. The term can be defined as that proportion of a micronutrient or bioactive compound that remains soluble within the intestine lumen following a simulated gastrointestinal digestive process, in which the physiological conditions of the stomach and small intestine are accurately replicated [8]. In vitro digestion models are cost-effective, easy to control, and independent of physiological effects. Furthermore, they exhibit excellent reproducibility and can provide a reliable approximation of in vivo digestion [1]. 

Nevertheless, the majority of studies on in vitro digestion to date have focused on fresh vegetables. As a result, there is a deficiency of knowledge regarding the impact of heat treatment on the bioaccessibility of phytochemicals. Therefore, given that Brussels sprouts are one of the richest sources of glucosinolates [9], the objective of this study was to assess the impact of heat treatment and in vitro digestion on the levels of selected bioactive compounds in Brussels sprouts. It was hypothesized that the type of thermal processing method could influence the level of glucosinolates, isothiocyanates, and indoles. Futhermore, it was assumed that the type of thermal treatment would affect the degree of digestion of the analyzed compounds and their final bioaccessibility.

## 2. Materials and Methods

### 2.1. Experimental Material

The analysis was conducted on Brussels sprouts procured from a local supermarket (Cracow, Poland) in 2019. A batch of Brussels sprouts (2 kg raw weight, in triplicate) was subjected to a preliminary cleaning process and then divided into four subsamples. Subsequently, three subsamples underwent hydrothermal treatment. These included traditional boiling (B; *n* = 3), steaming (S; *n* = 3), and sous-vide (SV; *n* = 3). Additionally, one sample was maintained in its raw state and was not subjected to thermal treatment (R; *n* = 3). The parameters of the hydrothermal treatment (time, temperature, and water ratio) were previously optimized based on color and sensorial analyses, dry matter, protein, fat, ash, total dietary fiber, and soluble fraction content [10,11]. All hydrothermal treatments were conducted until consumable softness was reached, as determined by texturometer EZ Test X (Schimadzu’s, Hertogenbosch, The Netherlands).

The conventional cooking method was employed using stainless steel dishes and an induction hob (Hendi, Hamburg, Germany). The cooking temperature was set at 98 ± 1 °C, and the cooking time was 15 min [6]. The ratio of raw material to water was 1:3 (m:V) and was maintained throughout the boiling process by covering the pot. This was performed to prevent water evaporation.

Steamed vegetables were prepared in a Retigo Orange Version 6 × GN1/1|O 611 in a combi steamer (Retigo, Rožnov pod Radhoštěm, Czech Republic) at 100 °C for 7 min. [6].

Subsequently, sous-vide cooking was conducted by vacuum packing the samples (using a vacuum packer, VBN-4, RM Gastro, Veselínad Lužnicí, Czech Republic) at 90 °C for approximately 45–50 min, utilizing the sous-vide 225–448 system (Hendi, Hamburg, Germany) [6]. Following the completion of this stage of the experiment, all samples were subjected to a process of cryogenic freezing at a temperature of −20 ± 2 °C (blast chilling chamber, RedFox SHS-511, RM Gastro, Veselínad Lužnicí, Czech Republic). Subsequently, the frozen vegetables were subjected to lyophilization, which was accomplished using a Christ Alpha 1-4 lyophilizer (Martin Christ Gefriertrocknungsanlagen GmbH, Osterode am Harz, Germany), and ground in a Tecator Knifetec 1095 laboratory mill (Foss Tecator, Uppsala, Sweden) in order to obtain a homogeneous material (particle size 30–150 µm). The material was stored under refrigeration (4 ± 1 °C) in hermetically sealed containers until it was utilized for chemical analyses.

### 2.2. Methods

#### 2.2.1. Dry Matter

The dry matter content was determined by the dry-weighing method according to PN-ISO 712:2002 [12] with the use of a Venticell 55Plus dryer (BMT, Brno, Czech Republic).

#### 2.2.2. In Vitro Digestion

The in vitro digestion procedure was conducted in accordance with the methodology originally presented by Żyła et al. [13], with modifications proposed by Starzyńska-Janiszewska et al. [14]. In detail, 0.5 g of homogenized plant material was placed in syringes, 1 mL of distilled water was added, and the contents were mixed. Subsequently, the solution was acidified to a pH of 2 with 0.5 M HCl, and 0.2 mL of pepsin solution (concentration 6 mg/mL; activity 3850 U/mg; Sigma-Aldrich P6887 (Merck KGaA, Darmstadt, Germany); dissolved in 0.1 M HCl) was added. Distilled water was then added (in order to obtain a sample-to-solution ratio of 1:4), and the resulting solution was incubated at 37 °C for 2 h (in vitro gastric digestion). After this, 1 M NaHCO_3_ was added in a volume providing a pH of 7 and 0.5 mL of a bile and pancreatin solution (bile and pancreatin concentrations of 90 mg/mL and 9 mg/mL, respectively; bile—The Sigma-Aldrich B8631 (Merck KGaA) was dissolved in 0.1 M NaHCO_3_ and pancreatin—Sigma-Aldrich P7545 (Merck KGaA); specific activity: 4 × USP specifications). The solution was then redissolved in redistilled water (in vitro intestinal digestion) and mixed before being transferred to dialysis bags (Sigma-Aldrich D9777-100FT, Merck KGaA, dialysis tubing cellulose membrane). The dialysis bags were then closed and placed in flasks containing the imidazole buffer solution (3.4 g of imidazole (Sigma-Aldrich 792527, Merck KGaA) dissolved in 250 mL of redistilled water, pH adjusted to 7.0; supplemented with 11.688 g of NaCl anhydrous and redistilled water to 2 L). Subsequently, the flasks were placed in a shaking water bath (GFL 1092; GFL, Burgwedel, Germany) and incubated for 2 h at 37 °C. The dialysates were frozen at −22 ± 1 °C, then immediately lyophilized and used for further analyses.

#### 2.2.3. Determination of Glucosinolates

The glucosinolates (GLS) were determined as desulfoglucosinolates according to the standard ISO 9167 procedure [15]. However, in order to accommodate for the modifications to this procedure proposed by Kusznierewicz et al. [16], a further modification to the identification of GLS was implemented. The freeze-dried plant material (0.2 g) was extracted on two separate occasions using boiling methanol (70%, 3 mL, 15 min).

**Figure 1:**
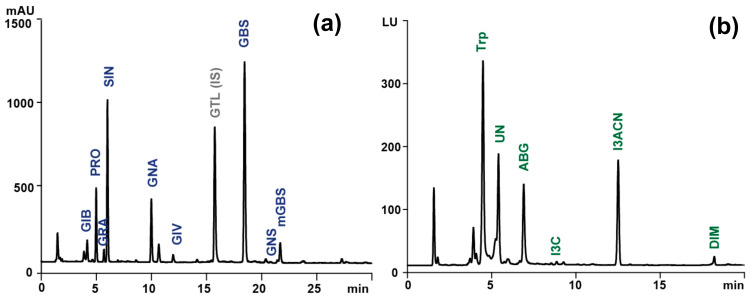
Sample chromatogram of (**a**) desulfoglucosinolates (λ = 229 nm) obtained during LC-PDA analysis and (**b**) indolic compounds (ex./em. −280/360 nm) obtained during LC-FLD analysis of extract from raw Brussels sprouts. GIB, glucoiberin; PRO, progoitrin; GRA, glucoraphanin; SIN, sinigrin; GNA, gluconapin; GIV, glucoiberverin; GTL (IS), glucotropaeolin (internal standard); GBS, glucobrassicin; GNS, gluconasturcin, metGBS, methoxyglucobrassicin; Trp, tryptophan; UN, unknown; ABG, ascorbigen; I3C, indole-3-carbinol; I3ACN, 3-indoleacetonitrile; DIM, diindolylmethane.

Subsequently, the supernatants were collected and combined after centrifugation (2800× *g*, 10 min, 4 °C). The extract, comprising approximately 6 mL, was then loaded onto a column containing 1 mL of a suspension of DEAE Sephadex A-25 anion exchange resin (Merck KGaA), which had been prewashed with 2 mL of imidazole formate (6 M) and twice with 1 mL of water. Then, the water solution of sulphatase (Helix pomatia type H1, Merck KGaA, 1 mg/mL, 250 µL) was added to the column. On the following day, the desulfo-glucosinolates (DS-GLS) were eluted with water (1 mL) and injected (30 µL) into the UltiMate 3000 UPLC system (Thermo Fisher Scientific, Waltham, MA USA), which consisted of a quaternary pump, well plate autosampler, and column compartment. The system was equipped with a SynergiTM Hydro-RP 80 Å column (150 × 4.6 mm, 4 μm, Phenomenex, Torrance, CA, USA) and a PDA detector, coupled with a high-resolution Thermo Q-ExactiveTM Focus quadrupole-Orbitrap mass spectrometer (Thermo Fisher Scientific). The mobile phase consisted of water (A) and a 20:80 (*v*/*v*) mixture of acetonitrile and water (B), both components of which contained 5 mM ammonium formate and 0. 01% (*v*/*v*) formic acid. The chromatographic resolution was conducted at 30 °C with a flow rate of 1 mL/min and a gradient program comprising a linear increase from 5 to 100% B over 25 min, followed by isocratic separation with 100% B for 5 min. The chromatographic peaks were initially detected with PDA at 229 nm, and then the identity of individual DS-GLS was confirmed via ESI-HRMS. The parameters employed in the negative ion acquisition mode of the HESI apparatus were as follows: sheath gas flow rate, 35 arb; auxiliary gas flow rate, 15 arb; sweep gas flow rate, 3 arb; spray voltage, 2.5 kV; capillary temperature, 350 °C; S-lens RF level, 50; and heater temperature, 300 °C. The full MS scan was configured as follows: resolution, 7 × 10^4^ FWHM; AGC target, 2 × 10^5^; maximum injection time, 100 ms; scan range, 120–1200 *m*/*z*. The data-dependent MS2 parameters were: resolution, 1.75 × 10^4^ FWHM; isolation window, 3.0 *m*/*z*; collision energy, 30 eV; AGC target, 2 × 10^5^; max inject time, 100 ms. The data were analyzed with Xcalibur 4.1 software (Thermo Fisher Scientific). An aqueous solution of glucotropaeolin (GTL) (5 mM, 0.2 mL) (AppliChem, Darmstadt, Germany) was added to each sample immediately prior to the initial extraction as an internal standard for the quantitative analysis, in accordance with the ISO protocol [15]. The resulting chromatographic and spectrometric data, along with the associated chromatograms, are presented in Table 1 and Figure 1.

#### 2.2.4. Determination of Indoles and Isothiocyanates

Brussels sprout freeze-dried extracts (0.4 g) were prepared in phosphate buffer (0.01 M, pH = 7.4, 10 mL) and then incubated for 3 h at 37 °C to allow for the potential action of endogenous myrosinase. For each sample, three independent extractions and analyses were conducted.

The obtained extracts were purified and enriched using the SPE (solid phase extraction) technique according to the conditions previously described [16,17]. An aliquot (10 mL) of water extracts was added to the Bakerbond C18 column (Octadecyl C_18_, 500 mg, 3 mL, J. T. Baker, Greisheim, Germany), which had been preconditioned with 3 mL of methanol and 3 mL of water. After sample loading, the stationary phase was dried under the stream of air (5 min). Then, the analytes were eluted with 1 mL of isopropanol. The obtained SPE eluate was divided into three portions.

A part of the SPE eluation was analyzed for indole content according to the methodology described by Pilipczuk et al. [4]. An Agilent 1200 Series HPLC-PDA-FLD system (Agilent Technologies, Santa Clara, CA, USA) equipped with Zorbax Eclipse XDB-C8 (150 × 4.60 mm, 3.5 μm) was employed for indole determination. The analytes were separated using a gradient elution method with 0.1% formic acid water (mobile phase A) and acetonitrile (mobile phase B) at a flow rate of 1 mL/min. The elution program was as follows: 0 min, 20% B; 15 min, 44% B; 22 min, 100% B; and 25 min, 100% B. The injection volume for the extracts was 20 μL. Indoles were monitored by UV detection at 280 nm (Agilent 1200 series diode array detector) and/or fluorescence detection at 280/360 (ex./em.) (Agilent 1260 fluorescence detector). The indolic contents in plant extracts were calculated using a calibration line of standard indole-3-carbinol (I3C) (Merck KGaA) and expressed as I3C equivalents (linear range, 0.02–10 nmol/mL; standard curve, y = 152.59x − 0.0033; r^2^ = 0.990; limit of detection (LOD), 0.001 nmol/mL; limit of quantification (LOQ), 0.003 nmol/mL).

The remaining two portions of the eluate were used to determine individual isothiocyanate (ITC) in the form of conjugates with N-acetyl-l-cysteine (NAC) and to determine the total content of ITC reacted with 1, 2-benzenedithiol (BDT) to 1, 3-benzenedithiole-2-thione (BDTT) according to the procedure and conditions described by Pilipczuk et al. [5]. The total content of ITC was determined by the method described previously by Zhang et al. [18]. The SPE eluate (0.1 mL) was added to the reaction mixture consisting of potassium phosphate buffer (0.5 mL, 0.1 M, pH 8.5), isopropanol (0.5 mL), and BDT in isopropanol (0.1 mL, 60 mM). After incubation (60 min, 65 °C), the mixture was analyzed for BDTT content with the Agilent 1200 system (Agilent Technologies) coupled with a PDA detector and Kinetex PFP column (150 mm × 4.6 mm, 5 μm, Phenomenex, USA). The volume of reaction mixture injected into the chromatographic system was 30 μL. The mobile phase contained 0.1% (*v*/*v*) formic acid in water (solvent A) and 0.1% (*v*/*v*) formic acid in acetonitrile (solvent B) and flowed at a rate of 1 mL/min. The gradient program was as follows: 0 min, 60% B, and 12 min, 100% B. Chromatograms were recorded at 365 nm. The calibration curve used for quantification of total ITC in plant samples was generated by the integration of the area of absorption peak of BDTT determined during analyses of serial dilutions of phenylethyl isothiocyanate standard (Merck KGaA) mixed and incubated with BDT (linear range, 0.01–0.4 μmol/mL; standard curve, y = 2956x + 9.25; r^2^ = 0.998; LOD, 0.002 µmol/mL; LOQ, 0.006 µmol/mL).

**Figure 2:**
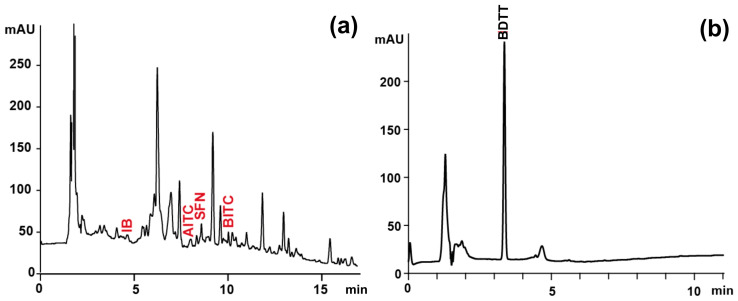
Sample chromatograms obtained during determination of (**a**) individual ITC as ITC-NAC conjugates (λ = 272 nm) and (**b**) total ITC as BDTT (λ = 365 nm) of extract from raw Brussels sprouts. IB, iberin; AITC, allyl-ITC; SFN, sulforaphane; BITC, 3-butenyl-ITC; BDTT, 1, 3-benzenedithiole-2-thione.

In the case of individual ITC determination, the third portion of the SPE eluate (500 μL) was mixed with a water solution of NAC (0.2 M, 250 μL) and NaHCO_3_ (0.2 M, 250 μL). The reaction mixtures were incubated for 1 h at 50 °C to accomplish the derivatization. After incubation, the samples were chilled and injected into the Ultimate 3000 UPLC system (Thermo Fisher Scientific) equipped with a PDA detector and Kinetex PFP column (150 mm × 4.6 mm, 5 μm, Phenomenex, Torrance, CA, USA). The solvents in the mobile phase were: 0.1% (*v*/*v*) formic acid in water (solvent A) and 0.1% (*v*/*v*) formic acid in acetonitrile (solvent B), with a flow rate of 1 mL/min. The linear gradient for solvent B was as follows: 0 min, 5%; 15 min, 40%; 20 min, 65%; 25 min 100%. An injection volume of 10 μL was used. The analytes were traced at 272 nm. The identity of individual ITC was confirmed via ESI-HRMS (Thermo Q-ExactiveTM Focus Quadrupole-Orbitrap mass spectrometer, Thermo Fisher Scientific) based on specific ions (Table 1, Figure 2). The HESI parameters in positive acquisition were the same as described for GLS determination. The quantification of the analytes was conducted using external calibration curves.

#### 2.2.5. Bioaccessibility

The bioaccessibility of glucosinolates, isothiocyanates, and indoles was calculated as the ratio of the amount of the ingredient that diffused through the cellulose membrane to the total amount of the ingredient in the starting material, according to the following formula [6]:

$$\mathrm{bioaccessibility}\;(\%)=\;\frac{\mathrm{bioaccessible}\;\mathrm{amount}\;\mathrm{of}\;\mathrm{the}\;\mathrm{component}\;\mathrm{from}\;\mathrm{vegetable}\;\mathrm{sample}\;(\mathrm{after}\;\mathrm{in}\;\mathrm{vitro}\;\mathrm{digestion})}{\mathrm{total}\;\mathrm{amount}\;\mathrm{of}\;\mathrm{the}\;\mathrm{component}\;\mathrm{from}\;\mathrm{vegetable}\;\mathrm{sample}\;(\mathrm{before}\;\mathrm{in}\;\mathrm{vitro}\;\mathrm{digestion})}\times 100\%$$

### 2.3. Statistical Analysis

The data were subjected to a statistical analysis. The hypothesis of the normality of the distribution of results was verified using the Shapiro–Wilk test, while the homogeneity of variance was verified with the Lavene’s test. The data that met the normality assumption, but lacked homogeneity of variance were subjected to Welch’s ANOVA, followed by Sheffe’s post-hoc test. In instances where the data exhibited a non-normal distribution, the Kruskal–Wallis test was employed. The significance of the results was determined through the application of a *p*-value cut-off of <0.05. All analyses were performed using the Statistica 13.0 software, developed by Stat-Soft Inc., Tulsa, OK, USA.

## 3. Results

Table 2 presents the content of GLS and their degradation products in the raw and thermally processed Brussels sprouts. The total glucosinolate content in the raw Brussels sprouts was found to be 15.05 µmol/g d.w. (dry weight), which equates to 239.7 µmol/100 g f.w. (fresh weight). The two principal glucosinates identified in the raw sample were aliphatic GLS sinigrin and indole GLS glucobrassicin, with levels of 4.78 µmol/g d.w. and 3.75 µmol/g d.w. (equivalent to 76.1 µmol/100 g f.w. and 59.8 µmol/100 g f.w., respectively). The total GLS content of the samples subjected to heat treatment ranged from 13.26 µmol/g d.w. (199.5 µmol/100 g f.w., sous vide) to 17.65 µmol/g d.w. (267.6 µmol/100 g f.w., conventional cooking).

The most abundant isothiocyanates in the raw material were 3-butenyl-ITC (BITC), followed by iberin, and 2-propenyl-ITC. All of these compounds were present at levels of approximately 0.3 µmol/g d.w., resulting in a total sum of ITC of 0.91 µmol/g d.w. The fourth ITC analyzed, sulforaphane (SFN), was identified in much lower amounts (0.035 µmol/g d.w.). With regard to indoles, only tryptophan (Trp), β-glucoside (BG), and indole-3-acetonitrile (I3ACN) were identified in the Brussels sprout samples. The total sum of indoles ranged from 0.106 µmol/g d.w. (steam) to 0.128 µmol/g d.w. (sous vide method).

The levels of the investigated compounds after simulated digestion (in dialysates) were generally lower, with some exceptions (Table 2). The total GLS content was reduced approximately tenfold, and some compounds were completely degraded. The levels of isothiocyanates analyzed by the Zhang method also decreased, while the content of indoles remained at the same level.

## 4. Discussion

### 4.1. Stability of Glucosinolates and Its Metabolites during Thermal Processing

The glucosinolate-myrosinase system in *Brassica* plants is susceptible to thermal changes at multiple levels due to heating or cooking. High temperatures can result in the complete or partial inactivation of myrosinase, the leaching of glucosinolates or their metabolites into the cooking medium, the loss of enzymatic cofactors, and the thermal degradation or volatilization of metabolites [19].

In the initial phase of the experiment, samples of Brussels sprouts were subjected to three distinct thermal treatments, including conventional cooking, steaming, and sous vide, and then analyzed to determine the content of glucosinolates and their metabolites. Eight glucosinolates were identified and quantified. These included six aliphatic methionine-derived compounds, two indolic tryptophan-derived compounds, and one aromatic phenylalanine-derived compound. The glucosinolates identified were: glucoiberin, progoitrin, glucoraphanin, sinigrin, gluconapin, and glucoibervein, as well as glucobrassicin and 4-methoxyglucobrassicin. Gluconaatrurtiin was identified as a single, aromatic phenylalanine-derived compound. The total GLS content and the proportions of the different GLSs in raw Brussels sprouts were found to be consistent with the data found in the literature [3,7,9]. However, it is important to note that the presence of GLSs is influenced by numerous additional factors, including soil characteristics, seasonal variations, genetic predisposition, and interactions between elements and anthropogenic pollution [20]. For example, in our previous study, the total GLS sum reached a comparable level (215 µmol/100 g f.w.), whereas Ciska et al. [21] reported a markedly higher GLS content in raw Brussels sprouts (498 µmol/100 g f.w.). Sinigrin and glucobrassicin were also identified as the most prevalent glucosinolates in Brussels sprouts in other studies [9,22].

The glucosinolate levels (GLS) of the samples exhibited significant variability in their susceptibility to thermal degradation. No statistically significant differences were found when comparing the total GLS results obtained from the heat-treated samples and in the raw product. However, the total glucosinolate content was observed to be statistically higher in the conventionally cooked product than in the sous vide method. Furthermore, variable behavior of the substances was observed under the influence of the different heat treatment methods, with the individual compounds exhibiting varying responses to the different heat treatment methods. In general, the content of the compounds remained unchanged after sous-vide treatment, with the exception of GNA and PRO, which decreased by 35% and 25%, respectively, and metGBS, which increased by 284%. The application of steaming resulted in a statistically significant reduction in the amount of GNA (by 20%) and an increase in the level of metGBS (by 423%). The conventional cooking resulted in an increased glucosinolate content, specifically GIB (117%), GBS (150%), and metGBS (437%). These findings are in accordance with those of a previous study [23], which demonstrated that cooking Brussels sprouts by the sous-vide method resulted in significantly higher losses of glucosinolates, both indole and aliphatic, compared to conventional cooking.

The increase in the levels of certain GLSs has already been documented by other researchers in the field. It is possible that the increase in total GLS content is attributable to thermal degradation of plant tissues, which may facilitate more effective chemical extraction of GLS from processed samples relative to raw material. This also suggests that the standard method, in which 70% methanol at 70 °C is used, does not support sufficient extraction of GLS from raw vegetables. Consequently, the GLS content of fresh vegetables may be underestimated [21]. Therefore, the results obtained by other researchers may differ significantly depending on the method used to prepare the samples. Previous research has indicated that boiling can result in a reduction in GLS content, with observed decreases of up to 90% [24]. However, in several experiments, thermal treatment of *Brassica* species by means of steaming, for instance, did not result in a notable reduction in glucosinolate concentrations [19].

The observed variation in loss has been attributed to the differing chemical structures and thermal labilities of aliphatic and indole glucosinolates [21]. However, the results of this experiment demonstrated no significant differences between aliphatic glucosinolates and the three other GLSs in terms of the observed changes during heat treatment.

Following disruption of the cell, glucosinolates can be converted by myrosinase into isothiocyanates and indoles, some of which have been demonstrated to possess beneficial properties for human health [25]. As an illustrative example, hydrolysis of glucobrassicin by plant or bacterial myrosinase results in the production of multiple indoles, predominantly indole-3-carbinol (I3C) [22]. Conversely, allyl ITC and 3-butenyl ITC are derived from sinigrin and gluconapin, respectively [9]. In order to achieve this, the content of the individual isothiocyanates and indoles was determined in all products, both the raw material and the product resulting from thermal processing. The presence of the four isothiocyanates analyzed by the ITC-NAC method was identified exclusively in the raw Brussels sprouts, which may suggest that they were degraded by the heat treatment or transferred to the water. In contrast, the ITC (expressed as total ITC content) analyzed by the Zhang method was detected in all products, including those subjected to heat processing. However, no statistically significant differences were observed in their levels compared to the raw product. Furthermore, no statistically significant differences in ITC values were observed between the various heat processing methods. The total ITC content obtained by this technique was comparable to the ITC-NAC method and was equal to 0.9 µmol/g d.w., which is in good agreement with the value reported by Sun et al. [26] of approximately 1 µmol/g d.w. Among the indoles, only three compounds were detected, and their levels were not statistically different.

Previous studies have documented changes in ITC during the processing of Brussels sprouts. It was observed that there was a relatively high abundance of ITC present in boiled Brussels sprouts [9]. Of these, the allyl ITC and 3-butenyl ITC were identified as the two most prevalent forms. In contrast, the presence of ITC was detected in a much lower abundance in steamed Brussels sprouts compared to the other cooking treatments. With regard to sulforaphane (SFN), one of the most health-promoting ITCs, its presence was not observed after any of the cooking methods. This observation has been previously noted [21,26,27]. According to the authors, the loss of SFN can be attributed to the heat inactivation of myrosinase, as well as the leaching of glucoraphanin (sulforaphane precursor) and sulforaphane into the boiling water. Additionally, it is possible that ITC could be volatilized during the cooking process.

### 4.2. Bioaccessibility of Phytochemicals after In Vitro Digestion

In order to evaluate the bioaccessibility of the bioactive compounds present in Brussels sprouts, samples that had undergone thermal treatment were subjected to a simulated in vitro digestion using gastric, bile, and pancreatic enzymes. The analysis of the glucosinolate content after digestion revealed a reduction in the levels of individual compounds following all thermal treatment variants, with values ranging from 1.25 µmol/g d.w. to 2.32 µmol/g d.w. This indicates that less than 15% of the GLS content was bioaccessible to human enterocytes.

To date, there has been limited research on the in vitro digestion of thermally processed Brussels sprouts. In a recent study, Vancoillie et al. [3] observed that the total amount of glucosinolates remained similar throughout the in vitro digestion of heated Brussels sprout puree. This resulted in the hypothesis that prior heating of vegetables could have a stabilizing effect on the majority of phytochemicals considered. However, there is a lack of data regarding the bioaccessibility ratio obtained for the sum as well as for the individual glucosinolates. Similar studies on other species of *Brassica* vegetables treated with simulated in vitro digestion have been conducted, primary on broccoli, and the results obtained were largely comparable, although the experiments were conducted exclusively on raw vegetable material. To provide an illustration, Vallejo et al. [28] observed a 69% reduction in total glucosinolate content in broccoli during in vitro gastric digestion, with an additional 12% loss postintestinal digestion. This resulted in a bioaccessible fraction of only 19% for glucosinolates in the human digestive tract. Fernández-León et al. [29] also observed a loss of approximately 69% (equivalent to 31% of bioaccessibility) in terms of total glucosinolate content in broccoli and cabbage following simulated in vitro digestion. These observations are consistent with the findings of the study conducted by Camara-Martos et al. [20], which demonstrated that over 30% of the glucosinolates initially present in leafy *Brassica* vegetables could reach human enterocytes. However, it is important to note that these results were obtained using raw plants. Consequently, when compared with the results obtained from the processed samples, it can be concluded that the heat treatment of *Brassica* vegetables, including Brussels sprouts, may reduce the bioaccessibility of GLS by approximately 50%.

The changes in the levels of the individual glucosinolates were evaluated. It was found that GRA, GIV, and GNS were completely degraded and undetected in the samples after digestion. MetGBS was the most resistant compound to digestion, with its level decreasing to 40% after simulated digestion via the sous vide method and 35% after steaming (Table 3). However, in the case of conventional cooking, in vitro digestion resulted in the complete degradation of metGBS.

With regard to the impact of thermal processing on glucosinolate bioavailability, statistically significant differences were observed only for GIB in conventionally cooked samples (significantly lower content after digestion, 9%) in comparison to steaming and sous vide methods. (21% and 27%, respectively) and for GNA in samples cooked by the sous-vide technique (significantly higher content after digestion, 23%) compared to steaming (only 6%). In conclusion, it can be stated that the digestion process is more effective after sous vide and traditional cooking methods, and to a lesser extent after steaming.

Unfortunately, there were no adequate reports in the literature for processed Brussels sprouts to enable comparison. According to Cuomo et al. [30], glucoraphanin from steamed broccoli was degraded by just 5% following in vitro digestion. A comparable experiment was performed with white cabbage (*Brassica oleracea* L. var. *capitata*), Chinese cabbage (*Brassica rapa* L. ssp. *pekinensis*), and bok choy (*Brassica rapa* L. ssp. *chinensis*), which were steamed, blanched, dried, and pulverized. Kuljarachanan et al. [2] demonstrated that glucoraphanin remained stable throughout the in vitro digestion process. These findings are contradictory to those of our study, in which glucoraphanin was completely degraded in all cases. Conversely, Rodríguez-Hernández et al. [31] conducted an in vitro digestion of different cultivars of raw freeze-dried broccoli and measured the glucoraphanin content, which decreased by only 7%. In a similar study, Fernández-León et al. [29] found that glucobrassicin exhibited the highest value of bioaccessibility in comparison to other glucosinolates present in raw broccoli and cabbage. The bioaccessibilities of the predominant glucosinolates were moderate, with values ranging from 13% for glucoraphanin to 43% for gluconapin. Hwang et al. [1] observed that immediately after 30–60 min of simulated gastric digestion, the levels of sinigrin, gluconastrurtiin, glubrasscin, and 4-methoxyglucobrassicin were significantly reduced compared to the levels present in fresh kale. After 120 min, the levels of all glucosinolates were reduced, and glucobrassicin was no longer detectable in the digesta. In simulated intestinal digestion, the levels of glucoraphanin, sinigrin, and gluconapin were reduced by 29%, 32%, and 43%, respectively, whereas the losses of gluconasturtiin (99%) and 4-methoxyglucobrassicin (81%) were even more pronounced. This finding is in partial agreement with the results of our own experiment, in which gluconasturtiin was completely degraded after in vitro digestion. However, as previously stated, these results were obtained only on raw material subjected to simulated in vitro digestion. When compared with our results, it can be suggested that heat treatment of certain *Brassica* vegetables may result in a notable decrease in glucoraphanin levels after in vitro digestion.

In the case of isothiocyanates and indoles, no significant differences were observed between the contents of the samples after thermal treatment and after digestion. The results obtained were found to be at comparable levels, indicating that the compounds, if present in the samples following the cooking process, remained stable throughout the in vitro digestion procedure.

The stability of ITCs during digestion is not well documented. In the available literature, similar experiments have been performed on broccoli, wherein up to 50% of the released sulforaphane could be collected in the bioaccessible dialyzed fraction. The content of sulforaphane in the digesta (across both in the gastric and intestinal phases) increased during the digestion of raw or 1 min steamed broccoli, although no reduction in glucoraphanin concentration was observed in the same digested samples. Other findings indicated that at the acidic pH of the gastric phase, indole-3-carbinol can form dimers and condensation products, including diindolylmethane, and indolo[3,2-b]carbazole [25]. Abellan et al. [32] observed a concentration in digestates of over 0.10 mg/100 g f.w. in only those derived from red radish sprouts. This result aligns with previously documented descriptions in the literature of the gastric digestion capacity to extract ITCs and indoles from *Brassicas* following ingestion. Furthermore, the low pH of the simulated gastric fluid (SGF) is thought to facilitate the formation of nitriles and epionitriles, rather than ITCs, due to the acidity of the fluid.

## 5. Conclusions

Cruciferous vegetables are a valuable source of bioactive compounds. However, there has been a deficiency of information regarding the extent to which these compounds remain accessible to the body following thermal treatment and digestion processes within the digestive tract. In the experiment, we hypothesized that the type of thermal processing method could influence the level of glucosinolates, isothiocyanates, and indoles. It was also assumed that the specific thermal treatment would impact the extent of the analyzed compounds’ digestion and their bioaccessibility. The final outcomes showed that the type of thermal treatment may result in significant losses in the case of glucosinolates, while the content of isothiocyanates and indoles exhibited minimal changes. Simulated in vitro digestion resulted in a considerable reduction in glucosinolate content, with only 15% of the compounds becoming accessible to the body. Conversely, isothiocyanates and indoles appeared to be resistant to digestion. To maintain the glucosinolate content of cruciferous vegetables, it is advisable to avoid prolonged heat treatment, such as prolonged cooking. The most effective method for preserving the bioactive compounds in Brussels sprouts is through steaming. The results of this study may contribute to a more comprehensive understanding of the changes in bioactive compounds within a simulated digestive tract, thereby improving the bioaccessibility of glucosinolates in human nutrition.

## Figures and Tables

**Table 1:** Chromatographic and spectrometric data obtained during LC-PDA-HRMS analyses of Brussels sprout extracts used for putative identification of selected secondary metabolites.

Compound Name	RT [min]	Molecular Formula	Precursor Ion (*m*/*z*)			Diagnostic Ions (*m*/*z*)
			Theoretical	Experimental	Δm (ppm)	
Glucosinolates			[M_DS-GLS_-H]^−^			[M_DS-GLS_-H-162]^−^
[(2S,3R,4S,5S,6R)-3,4,5-trihydroxy-6-(hydroxymethyl)oxan-2-yl] (1E)-4-methylsulfinyl-N-sulfooxybutanimidothioate (glucoiberin; GIB)	3.9	C_11_H_21_NO_10_S_3_	342.068123	342.068329	0.60	180.016 [C_5_H_10_NO_2_S_2_]^−^
[(2S,3R,4S,5S,6R)-3,4,5-trihydroxy-6-(hydroxymethyl)oxan-2-yl] (3R)-3-hydroxy-N-sulfooxypent-4-enimidothioate (Progoitrin; PRO)	4.2	C_11_H_19_NO_10_S_2_	308.080401	308.080780	1.23	146.027 [C_5_H_8_NO_2_S]^−^
[(2S,3R,4S,5S,6R)-3,4,5-trihydroxy-6-(hydroxymethyl)oxan-2-yl] (1E)-5-methylsulfinyl-N-sulfooxypentanimidothioate (glucoraphanin; GRA)	5.7	C_12_H_23_NO_10_S_3_	356.083772	356.084106	0.94	194.030 [C_6_H_12_NO_2_S_2_]^−^
[(E)-1-[(2S,3R,4S,5S,6R)-3,4,5-trihydroxy-6-(hydroxymethyl)oxan-2-yl]sulfanylbut-3-enylideneamino] sulfate (Sinigrin; SIN)	5.9	C_10_H_17_NO_9_S_2_	278.069836	278.070099	0.95	116.016 [C_4_H_6_NOS]^−^
[(2S,3R,4S,5S,6R)-3,4,5-trihydroxy-6-(hydroxymethyl)oxan-2-yl] (1E)-N-sulfooxypent-4-enimidothioate (gluconapin; GNA)	9.9	C_11_H_19_NO_9_S_2_	292.085486	292.085937	1.54	130.032 [C_5_H_8_NOS]^−^
[(2S,3R,4S,5S,6R)-3,4,5-trihydroxy-6-(hydroxymethyl)oxan-2-yl] (1E)-4-methylsulfanyl-N-sulfooxybutanimidothioate (glucoiberverin; GIV)	11.9	C_11_H_21_NO_9_S_3_	326.073208	326.073547	1.04	164.020 [C_5_H_10_NOS_2_]^−^
[(2S,3R,4S,5S,6R)-3,4,5-trihydroxy-6-(hydroxymethyl)oxan-2-yl] 2-(1H-indol-3-yl)-N-sulfooxyethanimidothioate (glucobrassicin; GBS)	18.4	C_16_H_20_N_2_O_9_S_2_	367.096385	367.096558	0.47	205.043 [C_10_H_9_N_2_OS]^−^
[(2S,3R,4S,5S,6R)-3,4,5-trihydroxy-6-(hydroxymethyl)oxan-2-yl] 3-phenyl-N-sulfooxypropanimidothioate (gluconasturtiin; GNS)	21.4	C_15_H_21_NO_9_S_2_	342.101136	342.101471	0.98	180.048 [C_9_H_10_NOS]^−^
[(2S,3R,4S,5S,6R)-3,4,5-trihydroxy-6-(hydroxymethyl)oxan-2-yl] 2-(4-methoxy-1H-indol-3-yl)-N-sulfooxyethanimidothioate (4-methoxyglucobrassicin; metGBS)	21.7	C_17_H_22_N_2_O_10_S_2_	397.106950	397.106735	0.54	235.054 [C_11_H_11_N_2_O_2_S]^−^
Isothiocyanates			[M_NAC-ITC_+H]^+^			[M_NAC-ITC_+H-NAC]^+^
1-isothiocyanato-3-methylsulfinylpropane (Iberin; IB)	4.6	C_5_H_9_NOS_2_	327.050699	327.049974	2.22	164.017 [C_5_H_10_NOS_2_]^+^
3-isothiocyanatoprop-1-ene (Allyl-ITC; AITC)	7.2	C_4_H_5_NS	263.052412	263.051697	2.72	100.022 [C_4_H_6_NS]^+^
1-isothiocyanato-4-methylsulfinylbutane (sulforaphane; SFN)	7.7	C_6_H_11_NOS_2_	341.066349	341.065643	2.07	178.035 [C_6_H_12_NOS_2_]^+^
4-isothiocyanatobut-1-ene (3-butenyl-ITC; BITC)	8.9	C_5_H_7_NS	277.068062	277.0674438	2.23	114.037 [C_5_H_8_NS]^+^
Indoles			[M+H]^+^			
(2S)-2-amino-3-(1H-indol-3-yl)propanoic acid (tryptophan; Trp)	4.5	C_11_H_12_N_2_O_2_	205.097703	205.097107	2.91	118.065 [C_8_H_8_N]^+^, 146.060 [C_9_H_8_NO]^+^
Unknown (UN)	5.4	C_14_H_15_NO_4_	262.107934	262.107330	2.30	130.065 [C_9_H_8_N]^+^, 118.065 [C_8_H_8_N]^+^
(3S,3aR,6aS)-3,6,6a-trihydroxy-6-(1H-indol-3-ylmethyl)-3,3a-dihydro-2H-furo [3,2-b] furan-5-one (ascorbigen; ABG)	6.9	C_15_H_15_NO_6_	306.097764	306.096924	2.74	130.065 [C_9_H_8_N]^+^
1H-indol-3-ylmethanol (indole-3-carbinol; I3C)	8.8	C_9_H_9_NO	148.076239	148.0765378	2.02	130.065 [C_9_H_8_N]^+^, 118.065 [C_8_H_8_N]^+^
2-(1H-indol-3-yl) acetonitrile (3-Indoleacetonitrile; I3ACN)	12.5	C_10_H_8_N_2_	157.076573	-	-	-
3-(1H-indol-3-ylmethyl)-1H-indole (3,3’-diindolylmethane; DIM)	18.2	C_17_H_14_N_2_	247.123523	-	-	-

**Table 2:** Content of glucosinolates, isothiocyanates, and indoles in examined Brussel sprouts.

Content [µmol/g d.w.] (Mean ± Standard Deviation, *n* = 3)							
Compound	Raw (R)	Sous Vide (SV)	Steam (S)	Boiled (B)	Sous Vide-Digested (SV-D)	Steam-Digested (S-D)	Boiled-Digested (B-D)
Glucosinolates							
GIB *	0.736 ^ab^ ± 0.016	0.674 ^a^ ± 0.036	0.774 ^b^ ± 0.025	0.861 ^c^ ± 0.032	0.183 ^d^ ± 0.012	0.162 ^d^ ± 0.002	0.080 ^e^ ± 0.010
PRO *	2.162 ^ab^ ± 0.193	1.639 ^c^ ± 0.221	1.925 ^ac^ ± 0.154	2.542 ^b^ ± 0.293	0.088 ^d^ ± 0.027	0.259 ^d^ ± 0.012	0.278 ^d^ ± 0.023
GRA **	0.295 ^a^ ± 0.018	0.244 ^a^ ± 0.03	0.352 ^a^ ± 0.062	0.368 ^a^ ± 0.045	<LOD	<LOD	<LOD
SIN *	4.779 ^a^ ± 0.336	4.196 ^a^ ± 0.451	4.495 ^a^ ± 0.187	4.966 ^a^ ± 0.443	0.398 ^b^ ± 0.025	0.587 ^b^ ± 0.019	1.070 ^b^ ± 0.008
GNA *	2.795 ^a^ ± 0.152	1.799 ^b^ ± 0.196	2.235 ^c^ ± 0.060	2.493 ^ac^ ± 0.170	0.110 ^d^ ± 0.023	0.520 ^e^ ± 0.021	0.162 ^de^ ± 0.007
GIV **	0.304 ^ab^ ± 0.014	0.257 ^a^ ± 0.021	0.381 ^b^ ± 0.038	0.335 ^ab^ ± 0.056	<LOD	<LOD	<LOD
GBS *	3.753 ^a^ ± 0.059	4.074 ^a^ ± 0.781	4.792 ^ab^ ± 0.407	5.519 ^b^ ± 0.398	0.352 ^c^ ± 0.010	0.638 ^c^ ± 0.007	0.100 ^c^ ± 0.037
GNS **	0.119 ^ab^ ± 0.015	0.076 ^a^ ± 0.011	0.191 ^b^ ± 0.046	0.107 ^a^ ± 0.031	<LOD	<LOD	<LOD
metGBS **	0.105 ^a^ ± 0.037	0.299 ^b^ ± 0.031	0.444 ^c^ ± 0.052	0.460 ^c^ ± 0.057	0.120 ^a^ ± 0.012	0.153 ^a^ ± 0.021	<LOD
Total *	15.049 ^ab^ ± 0.425	13.259 ^a^ ± 1.670	15.589 ^bc^ ± 0.446	17.651 ^c^ ± 1.176	1.251 ^d^ ± 0.023	2.319 ^d^ ± 0.035	1.689 ^d^ ± 0.032
Isothiocyanates							
IB	0.298 ± 0.036	<LOQ	<LOQ	<LOQ	<LOQ	<LOQ	<LOQ
AITC	0.248 ± 0.007	<LOQ	<LOQ	<LOQ	<LOQ	<LOQ	<LOQ
SFN	0.035 ± 0.001	<LOQ	<LOQ	<LOQ	<LOQ	<LOQ	<LOQ
BITC	0.332 ± 0.025	<LOQ	<LOQ	<LOQ	<LOQ	<LOQ	<LOQ
Total	0.914 ± 0.019	<LOQ	<LOQ	<LOQ	<LOQ	<LOQ	<LOQ
Isothiocyanates by Zhang method							
Total ITC **	0.899 ^a^ ± 0.061	0.216 ^abc^ ± 0.087	0.093 ^abc^ ± 0.012	0.216 ^abc^ ± 0.057	0.078 ^abc^ ± 0.008	0.065 ^bc^ ± 0.003	0.061 ^bc^ ± 0.005
Indoles							
Trp **	0.044 ^a^ ± 0.003	0.033 ^a^ ± 0.002	0.038 ^a^ ± 0.006	0.036 ^a^ ± 0.009	0.035 ^a^ ± 0.007	0.042 ^a^ ± 0.014	0.034 ^a^ ± 0.001
UN **	0.021 ^a^ ± 0.001	0.009 ^ab^ ± 0.002	0.004 ^ab^ ± 0.001	0.007 ^ab^ ± 0.001	0.006 ^ab^ ± 0.001	0.003 ^ab^ ± 0.001	0.002 ^b^ ± 0.001
ABG **	0.018 ± 0.001	<LOD	<LOD	<LOD	<LOD	<LOD	<LOD
I3C	<LOD	<LOD	<LOD	<LOD	<LOD	<LOD	<LOD
I3ACN **	0.025 ^a^ ± 0.002	0.085 ^ab^ ± 0.003	0.063 ^ab^ ± 0.002	0.071 ^ab^ ± 0.005	0.093 ^b^ ± 0.003	0.067 ^ab^ ± 0.011	0.071 ^ab^ ± 0.014
DIM	<LOD	<LOD	<LOD	<LOD	<LOD	<LOD	<LOD
Total *	0.109 ^a^ ± 0.007	0.128 ^a^ ± 0.008	0.106 ^a^ ± 0.007	0.115 ^a^ ± 0.005	0.134 ^a^ ± 0.010	0.114 ^a^ ± 0.026	0.108 ^a^ ± 0.015

LOD—level of detection; GIB—Glucoiberin; PRO—Progoitrin; GRA—Glucoraphanin; SIN—Sinigrin; GNA—Gluconapin; GIV—Glucoiberverin; GBS—Glucobrassicin; GNS—Gluconasturtiin; metGBS—Methoxyglucobrassicin; IB—Iberin; AITC—Allyl-ITC; SFN—Sulforaphane; BITC—3-Butenyl-ITC; Trp—Tryptophan; UN—Unknown; ABG—Ascorbigen; I3C—Indole-3-carbinol; I3ACN—3-Indoleacetonitrile; DIM—3, 3’-Diindolylmethane. The same superscript letters in a line after mean values indicate no statistically significant differences between results (*p* > 0.05). * Welch’s Anova with Sheffe’s post-hoc test. ** Kruskal–Wallis test.

**Table 3:** The bioaccessibility of glucosinolates, isothiocyanates, and indoles after in vitro digestion.

Bioaccessibility [%]			
Compound	Sous-Vide Digested (SV-D)	Steam-Digested (S-D)	Boiled-Digested (B-D)
Glucosinolates			
GIB	30	41	41
PRO	25	35	41
GRA	27	46	43
SIN	29	37	36
GNA	21	31	31
GIV	28	49	38
GBS	36	50	51
GNS	21	63	31
metGBS	94	164	152
Total	29	40	41
Isothiocyanates by Zhang method			
Total ITC	8	4	8
Indoles			
Trp	25	34	28
UN	14	8	11
ABG	2	1	3
I3C	0	0	0
I3ACN	112	99	98
DIM	0	0	0
Total	38	38	36

GIB—Glucoiberin; PRO—Progoitrin; GRA—Glucoraphanin; SIN—Sinigrin; GNA—Gluconapin; GIV—Glucoiberverin; GBS—Glucobrassicin; GNS—Gluconasturtiin; metGBS—Methoxyglucobrassicin; Trp—Tryptophan; UN—Unknown; ABG—Ascorbigen; I3C—Indole-3-carbinol; I3ACN—3-Indoleacetonitrile; DIM—3, 3’-Diindolylmethane.

## Data Availability

The original contributions presented in the study are included in the article, further inquiries can be directed to the corresponding author.
